# CEP55 Positively Affects Tumorigenesis of Esophageal Squamous Cell Carcinoma and Is Correlated with Poor Prognosis

**DOI:** 10.1155/2021/8890715

**Published:** 2021-05-18

**Authors:** Shu-Mei Yan, Lili Liu, Wan-Yi Gu, Li-Yun Huang, Yi Yang, Yu-Hua Huang, Rong-Zhen Luo

**Affiliations:** ^1^Sun Yat-Sen University Cancer Center, State Key Laboratory of Oncology in South China, Collaborative Innovation Center for Cancer Medicine, Guangzhou 510060, China; ^2^Department of Pathology, Sun Yat-Sen University Cancer Center, Guangzhou, China; ^3^Department of Pathology, The Second Affiliated Hospital of Guangzhou Medical University, Guangzhou, China; ^4^Boao Evergrande International Hospital, Hainan, China

## Abstract

Centrosomal protein 55 (CEP55) is a centrosome- and midbody-associated protein that is overexpressed in several cancers. However, the underlying molecular mechanism of CEP55-mediated progression and metastasis of esophageal squamous cell carcinoma (ESCC) is not clear. In the current study, we detected CEP55 mRNA by qRT-PCR while protein expression was detected by western blot analysis and immunohistochemistry (IHC). In addition, we knocked down CEP55 and investigated the ability of CEP55 to affect colony formation and migration. Here, we report that CEP55 mRNA and protein expression was significantly increased in ESCC. IHC staining showed that CEP55 expression correlated with TNM stage (*p*=0.046) and lymph node metastases (*p*=0.024). According to overall survival (OS) and disease-free survival (DFS), patients whose tumors expressed a higher level of CEP55 had a poorer prognosis than those with low expression level of CEP55. A multivariate analysis revealed that CEP55 expression was an independent prognostic indicator for patients with ESCC. Knockdown of CEP55 decreased the colony formation ability and migration of ESCC cells and also reduced the phosphorylation of Src, FAK, and ERK. Therefore, our study implied that CEP55 may be a valuable biomarker and a potential target in the treatment of patients with ESCC.

## 1. Introduction

Human esophageal carcinoma is the sixth most common and lethal cancer in the world [1–3]. Squamous cell carcinoma (SCC) is one type of esophageal cancers that is frequently seen in Asia and Eastern Europe. One particular region has been termed the “Asian Esophageal Cancer Belt,” because the incidence of esophageal cancer exceeds 100/100000 per year [2, 4]. The National Comprehensive Cancer Network (NCCN) guidelines have suggested the use of preoperative chemoradiation therapy. Although some studies have shown that this combination therapy helps to decrease the 3 year mortality rate and locoregional recurrence [5, 6], the 5 year overall survival has remained unsatisfactory [7–9]. This is mainly due to the aggressiveness and malignancy of this cancer type. Hence, targeted therapies have become promising for the treatment of ESCC.

CEP55 (also known as c10orf3 and FLJ10540) is a centrosome- and midbody-associated protein that is critical for cell cycle progression and cytokinesis [10]. CEP55 is a highly coiled-coil protein that contains a hinge region, which has been termed the endosomal sorting complex; this complex is required for transport (ESCRT) and for the function of the ALIX-binding region (EABR) [11–13]. Over the past decade, many studies have demonstrated that CEP55 plays an important role in the cell cycle and in cell survival through regulation of the PI3K/AKT pathway [14, 15]. CEP55 also interacts with P110, which is mediated by VEGF-A [16]. Moreover, CEP55 maintains its stability and promotes increased levels of PIP3, which further increases AKT phosphorylation of S473 [17]. Currently, CEP55 has been found to be significantly associated with aggressiveness, TNM stage, and poor survival rate across various cancers, including breast [18], colorectal [19], and gastric carcinoma [15]. However, the way in which CEP55 influences tumorigenesis, migration, and prognosis in ESCC remains unknown.

Therefore, to explore the function of CEP55 in ESCC, in this study, we used quantitative reverse transcriptase PCR (qRT-PCR), immunohistochemistry (IHC), and western blot analysis to detect the mRNA and protein levels of CEP55 in ESCC tissues. We also analyzed the relationship between CEP55 expression and clinicopathological characteristics of patients with ESCC. Moreover, functional experiments including colony formation and migration assays were performed to show that the downregulation of CEP55 leads to the inhibition of colony formation and metastasis and increases the phosphorylation of Src, FAK, and ERK. This evidence demonstrates that CEP55 may be an emerging prospect for novel therapeutic strategies.

## 2. Materials and Methods

### 2.1. Patients and Tissue Specimens

This study was approved by the medical ethics committee of Sun Yat-sen University Cancer Center. The eleven ESCC biopsy specimens and adjacent paired normal esophageal mucosa tissues that were used for qRT-PCR and the five pairs of tissues that were used for western blot were collected from the Sun Yat-sen University Cancer Center (SYSUCC). These tissues were frozen in liquid nitrogen and stored throughout 2014. In all, 241 specimens from ESCC patients who underwent surgery were sectioned and confirmed by pathologic review of the IHC analysis after the exclusion of the noninformative samples (e.g., unrepresentative samples, samples with too few tumor cells and lost samples). All of the ESCC patients who were seen from October 2000 to April 2007 at the Sun Yat-sen University Cancer Center were histologically and clinically diagnosed and were treated with radical surgery without neoadjuvant/adjuvant treatments. The histologic grade and the clinical stage of the tumors were recorded based on the seventh edition of the TNM classification of the International Union against Cancer (2009) [20]. The selection criteria in this study are as follows: (1) newly diagnosed ESCC without previous treatment; (2) histologically confirmed primary ESCC; (3) no history of familial malignancies or other synchronous malignancies (e.g., gastrointestinal stromal tumor, gastric cancer, or colorectal cancer); (4) underwent resection plus lymphadenectomy (limited or extended) for esophageal cancer; (5) clinical information and follow-up data were available. Clinical data were obtained from hospital records after surgery. All of the patients were contacted in May 2012 to determine their vital status.

### 2.2. Tissue Microarray Construction

The tissue microarray was constructed according to methods that were described previously [21]. Tumor tissue specimens from 265 primary ESCC cases were collected, fixed in ethanol, and embedded in paraffin. Hematoxylin and eosin-stained sections from a single random block from each patient were reviewed by a senior pathologist (S.-M. Y.) to define representative tumor regions. Two targeted core samples of each specimen were obtained using a tissue array instrument (MiniCore instruments; Alphelys, Plaisir, France). Briefly, tissue cylinders with a diameter of 10 mm were punched and arrayed on a recipient paraffin block. Sections (5 *μ*m thick) of the tissue array (recipient) block were cut and placed onto glass slides.

### 2.3. Immunohistochemical Staining and Assessment

Tissue microarray sections that were deparaffinized and rehydrated by means of a graded alcohol series were used for the immunohistochemistry experiments. The slides were incubated in 0.3% hydrogen peroxide for 15 minutes to quench the endogenous peroxidase activity. For antigen retrieval, the tissue microarray slides were boiled in Tris (hydroxymethyl) aminomethane-EDTA buffer (pH 8.0) in a pressure cooker for 3 minutes. Sections were incubated with 10% normal goat serum for 10 minutes to block any nonspecific binding. The tissue microarray slides were then incubated with rabbit anti-human polyclonal antibody against CEP55 (1 : 1000 dilution, Abgent, San Diego, USA) for 12 hours at 4°C in a moist chamber. Blocking solution without the primary antibody was used as a negative control. After incubation with horseradish peroxidase for 30 minutes at 37°C, the slides were incubated with 3, 30-diaminobenzidine solution for visualization. Mayer's hematoxylin was applied as a counterstain. The negative control was obtained by replacing the primary antibody with normal murine immunoglobulin G. Positive expression of CEP55 in ESCC and normal esophageal mucosa cells was defined as a primarily nuclear staining pattern. When available, internal positive and negative controls, including normal squamous mucosa of the esophagus from cancer-free patients, were used to further support the staining patterns. Two independent observers (R. -Z. L. , M. L.), who were blinded to the clinicopathologic data, generated the immunoreactivity score for CEP55 expression. The staining results were scored based on the following criteria: (1) percentage of positive tumor cells in the tumor tissue: zero (0%), 1 (1%–10%), 2 (11%–50%), 3 (51%–80%), and 4 (71%–100%); and (2) signal intensity: zero (no signal), 1 (weak), 2 (moderate), and 3 (marked). The immunoreactivity score was calculated by multiplying the score for the percentage of positive cells by the intensity score (range, 0 to 12). The average immunoreactivity score for each case was designated as the staining result for the patient. The specimens were rescored if the difference between the scores as determined by the two pathologists was greater than 3.

### 2.4. Human Esophageal Cancer Cell Lines and Culture

The human esophageal cancer cell lines KYSE 520, KYSE 510, KYSE 30, Eca109, and KYSE 410 were obtained from Professor Xin-Yuan Guan (Department of Clinical Oncology, the University of Hong Kong). All cell lines were cultured in Dulbecco's Modified Eagle's Medium (DMEM) (Invitrogen) supplemented with 10% fetal bovine serum (FBS; Hyclone, Logan, UT). All cells were maintained at 37°C in a humidified incubator with 5% CO_2._

### 2.5. SiRNA Transfection

The two siRNAs against CEP55 were synthesized by Ribobio Technology (Guangzhou, China). The two sequences were as follows: siRNA^#^1: GAAGCCTAGTAACTCCAAA; siRNA^#^3: GGAAACAGCTGCTCATTCA.

The control mock siRNA was designated as siNC and was not homologous with any human genomic sequences. The KYSE 520 and Eca109 cells were transfected with 50 nM of the siRNA and 7.5 *μ*L of Lipofectamine RNAiMAX (Invitrogen) in six-well culture dishes, according to the manufacturer's instructions. The cells were incubated for 48 h and then harvested for further experiments.

### 2.6. qRT-PCR Analysis of mRNA

TRIzol reagent (Invitrogen) was used to isolate the total RNA from the tissue samples according to the manufacturer's protocol. The concentration and quality of the extracted RNA were analyzed by a NanoDrop spectrophotometer (ND-1000, Thermo Scientific, MA, USA). In all, 2 *μ*g of the total RNA from each sample was used for cDNA synthesis by a reverse transcriptase kit (Invitrogen). The primer sequences were as follows: CEP55 sense 5′-AGTAAGTGGGGATCGAAGCCT-3′,

CEP55 antisense 5′-CTCAAGGACTCGAATTTTCTCCA-3′, *β*-actin sense 5′-CGCGAGAAGATGACCCAGAT-3′, *β*-actin antisense 5′-GGGCATACCCCTCGTAGATG-3′.

qRT-PCR was performed with SYBR Green qPCR SuperMix-UDG (Invitrogen). All reactions were performed in triplicate in an ABI Prism-7500 Sequence Detector System (ABI, Applied Biosystems, Carlsbad, USA). *β*-actin served as an internal control. The relative expression of CEP55 mRNA was normalized to the expression of *β*-actin using the ΔΔCt method.

### 2.7. Western Blotting Assay

The protein concentration of the tissue samples and cells was measured by the BCA method using a protein assay kit. Proteins were separated on a 9% SDS polyacrylamide gel by electrophoresis (PAGE) and were then transferred to a polyvinylidene difuoride (PVDF) membrane (Pall, Port Washington, USA). The membrane was blocked with Tris-buffered saline plus Tween-20 (1x TBST) containing 5% BSA for 1 h. The membrane was incubated with the primary antibodies overnight at 4°C. Then, the membrane was washed three times with 1x TBST and incubated with the secondary antibody for 45 min at room temperature. The primary antibodies were as follows: anti-CEP55 (1 : 1000 dilution, Abgent, USA), anti-*β*-actin (1 : 3000 dilution, Protech, China), anti-phospho-FAK (D20B1) (Y397), anti-FAK, anti-phospho-ERK (T202) (Y204), anti-ERK, anti-phospho-Src, and anti-Src (1 : 1000 dilution, CST, Danvers, Massachusetts, USA).

### 2.8. Colony-Formation Assay

KYSE520 and Eca109 cells (1000 cells per well) were plated in 6-well plates and cultured for 10 days. The colonies were fixed in methanol for 15 min and then stained with 0.5% crystal violet in 20% methanol. All experiments were performed three times.

### 2.9. Transwell Assay

The cell migration assay was designed to examine the ability of the cells to migrate through a Transwell filter (BD Biosciences, San Jose, USA). KYSE520 (5 × 10^4^ cells) and Eca109 (1 × 10^5^ cells) cells were transfected and cultured for another 48 h. Then, the cells in the upper chamber were incubated with serum-free DMEM medium; DMEM with 10% FBS was added to the lower chamber. After the cells were cultured for 24 h, the cells that had migrated from the upper part of the chamber to the lower side of the chamber were fixed, stained, and counted under a microscope.

### 2.10. Statistical Analysis

To analyze the relationship of CEP55 mRNA expression with ESCC patients, the expression values of CEP55 in datasets were retrieved from the oncomine database (http://www.oncomine.org). Statistical analysis was performed with SPSS software (standard version 16.0, SPSS, Chicago, IL). The receiver operating characteristic method was used to define the cutoff value for the CEP55 immunoreactivity score. The receiver operating characteristic (ROC) curve was generated and analyzed using the MedCalc statistical software package 11.0.1 (MedCalc Software bvba, Mariakerke, Belgium). The correlation between CEP55 expression and clinicopathologic features of the patients with ESCC was assessed by Pearson's *X*^2^ test. A binary logistic regression model was used to analyze the variables that correlated with CEP55 expression. Disease-free survival (DFS) was defined as the time from surgery to regional relapse or the development of distant metastasis. Overall survival (OS) was defined as the time from surgery to death. Disease-free survival and OS were assessed with the Kaplan-Meier method and were compared by the log-rank test. A multivariate survival analysis was performed for all of the variables that were significant in the univariate analysis using the Cox regression model. A two-sided probability value of less than 0.05 was considered statistically significant.

## 3. Results

### 3.1. Expression of CEP55 Is Amplified in Human ESCC Tissues

**Figure 1:**
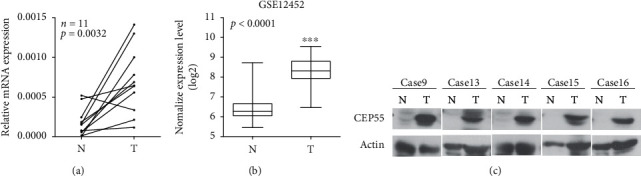
Expression of CEP55 is increased in ESCC samples and cell lines. CEP55 mRNA expression in 11 pairs of ESCC samples and adjacent nontumor tissues (a). 53 pairs samples data from oncomine data base (b). (c) CEP55 protein expression in ESCC samples (T) and matched normal tissues (N) by western blotting assay. N, matched noncancerous tissue; T, tumor tissue; ESCC, esophageal squamous cell cancer.

To determine the expression of CEP55 in ESCC tissues, qRT-PCR and western blot analyses were performed in matched ESCC tissues and adjacent noncancerous tissues. The level of CEP55 mRNA in the tumor tissues was significantly higher compared with that in the corresponding nontumor tissues by quantitative analysis (Figure 1(a)). The Oncomine database (http://www.oncomine.org) provided data on 53 pairs of ESCC samples and showed a similar result, which further demonstrated CEP55 overexpression in tumors (Figure 1(b)). Moreover, the expression of CEP55 protein was also higher in tumor tissues than in adjacent normal tissues (Figure 1(c)). In summary, the expression levels of both CEP55 mRNA and protein were upregulated in ESCC tissues.

### 3.2. Expression of CEP55 in ESCC by Immunohistochemistry

**Figure 2:**
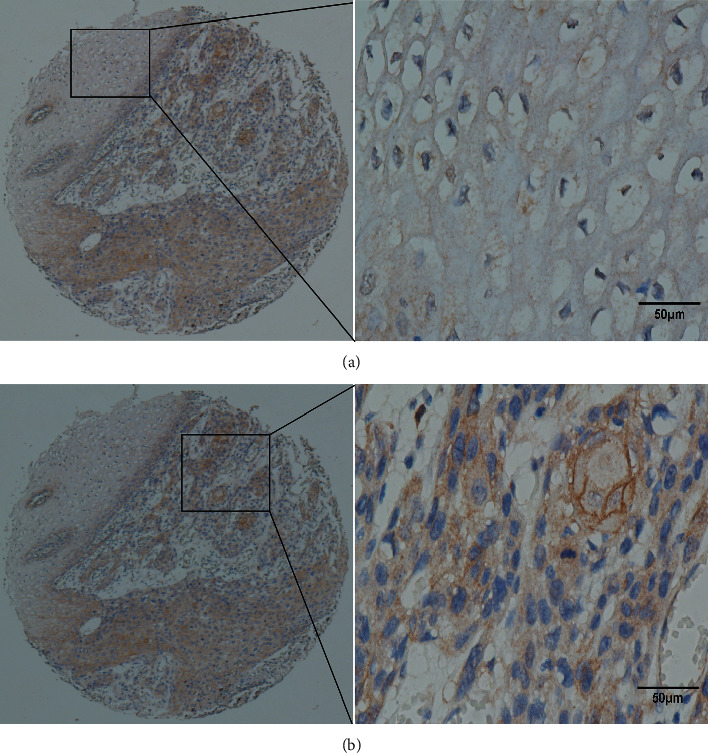
Representative staining of CEP55 in ESCC and pair normal tissues. CEP55 staining in the cytoplasm in tumor location was positive (a), but there was no faint in pair neighboring normal tissue (b).

Paraffin-embedded ESCC tissues were collected to detect CEP55 expression (*n* = 265). CEP55 was mainly expressed in the cytoplasm of ESCC cancer cells. The CEP55 IHC score for ESCC tissue was significantly higher than normal tissue (*p* < 0.001, Figure 2). In terms of CEP55 staining of ESCC and adjacent nonmalignant esophageal mucosal tissues, positive staining for CEP55 protein was primarily observed in the cytoplasm within ESCC cells (Figure S1).

### 3.3. Relationships between CEP55 Expression and Clinicopathologic Variables

We used the ROC curve to define the cutoff value for the CEP55 expression, as shown in Figure S2. High CEP55 expression was hence set as 8. The relationships between the clinicopathologic features of patients with ESCC and CEP55 expression are summarized in Table 1. A high level of CEP55 expression was found associated with node metastasis (*p*=0.024). ESCC patients with high levels of CEP55 expression had a higher proportion of late TNM stage (*p*=0.0046), as shown in Table 1.

### 3.4. Association of CEP55 Expression with Clinical Outcomes in ESCC Patients

**Figure 3:**
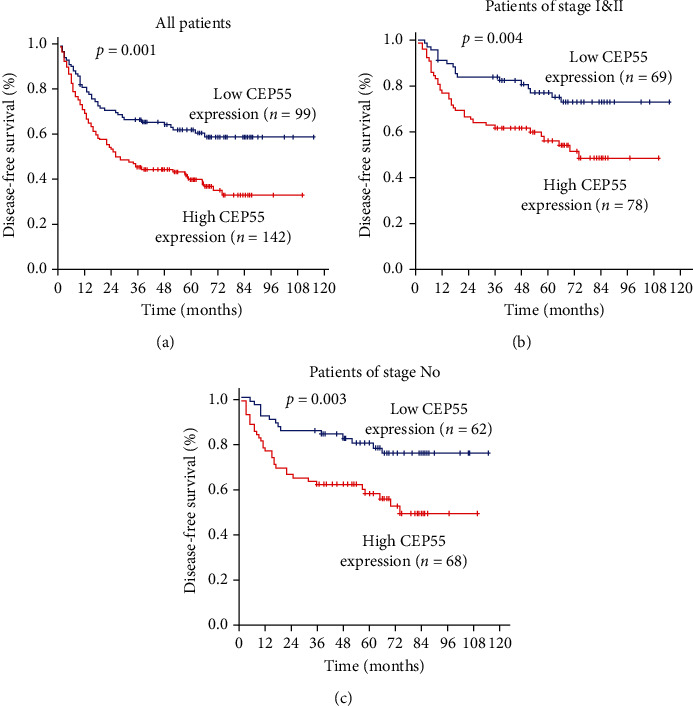
Kaplan–Meier analysis of prognosis of overall survival. (a) ESCC patients with high expression of CEP55 had significantly worse outcomes in terms of overall survival (*p* < 0.001). CEP55 could thoroughly distinguish OS when the patients were stratified by pN0 status (*p*=0.003), (b) and by TNM stage (*p*=0.003) (c).

CEP55 expression in ESCC was closely related to disease-free survival (DFS) and overall survival (OS) according to results of the Kaplan-Meier analysis. For patients with high expression of CEP55, results revealed that these ESCC patients had significantly worse outcomes in terms of overall survival (*p* < 0.001). To further confirm the potential prognosis prediction value of CEP55 in OS, we conducted subgroup analysis. CEP55 could thoroughly distinguish OS when the patients were stratified by pN0 status (*p*=0.003) and by TNM stage (*p*=0.003), as shown in Figure 3.

**Figure 4:**
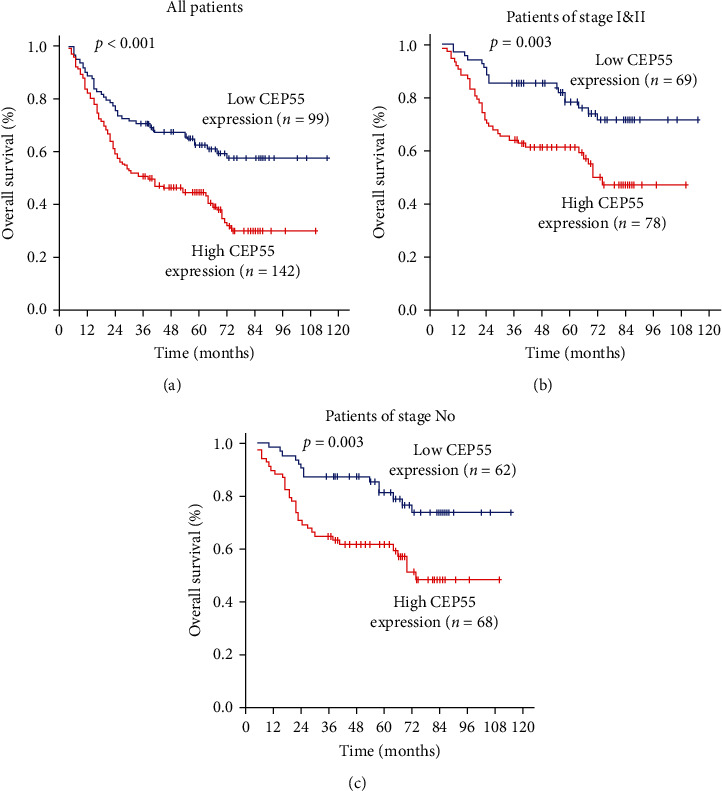
Kaplan–Meier analysis of prognosis of disease-free survival. (a) ESCC patients with low CEP55 expression had significantly better disease-free survival outcome than those with high CEP55 expression (*p*=0.001). CEP55 could distinguish DFS when the patients were stratified by pN0 status (*p*=0.003) (b) and by TNM stage (*p*=0.004) (c).

Similar trends were observed for disease-free survival, which showed that ESCC patients with low CEP55 expression had significantly better disease-free survival outcome than those with high CEP55 expression (*p*=0.001). Subgroup analysis was also conducted. CEP55 could distinguish DFS when the patients were stratified by pN0 status (*p*=0.003) and by TNM stage (*p*=0.004), as shown in Figure 4.

### 3.5. Univariate and Multivariate Analyses of Prognostic Variables in ESCC

To evaluate whether CEP55 expression was an independent risk factor for outcomes in ESCC, both univariate and multivariate analyses were conducted. pT status, N categorises, TNM stage, and CEP55 expressions were all shown to be prognostic variables for overall survival in ESCC patients. In the multivariate analysis, only pT status (*p* < 0.001), N categories (*p* < 0.001), and CEP55 expression (*p*=0.009) were found to be independent prognostic variables for overall survival (Table 2).

We also conducted analysis to evaluate risk factors associated with DFS. The results are shown in Table 3. pT status, N categories, TNM stage, and CEP55 expression were all shown to be prognostic variables for DFS in ESCC patients. In the multivariate analysis, only pT status (*p* < 0.001), N categories (*p* < 0.001). and CEP55 expression (*p*=0.015) were found to be independent prognostic variables for DFS (Table 3).

### 3.6. Upregulation of CEP55 Promotes Proliferation in ESCC Cells

**Figure 5:**
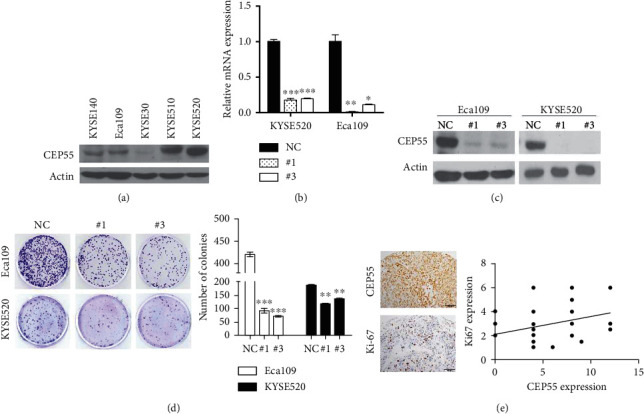
Downregulation of CEP55 inhibits the proliferation of ESCC cell lines. (a) CEP55 expression level in ESCC cell lines of KYSE140, Eca109, KYSE30, KYSE510, and KYSE520. (b) CEP55 RNA level in cell lines of Eca109 and KYSE520 cell after transfected with CEP55-RNAi. (c) Western blotting assay showed protein of CEP55 was successfully downregulated. (d) Downregulation of CEP55 reduced colony formation in ESCC cell lines not only sizes but also numbers. (e) A significant correlation was observed between Ki-67 expression and CEP55 in ESCC tissues (*r* = 0.28, *p*=0.049). ^∗∗^*p* < 0.01, ^∗∗∗^*p* < 0.001.

To investigate the function of CEP55 in ESCC, we knocked down CEP55 expression by transfection with siRNAs in ESCC cells. Among all ESCC cell lines, high expression of CEP55 was found in most of the cell lines especially of KYSE520 and Eca109 (Figure 5(a)). The mRNA level and protein level of CEP55 were downregulated after transfected with siRNA was confirmed (Figures 5(b) and 5(c)). We determined the cell proliferation by colony formation and found that both the numbers and sizes of the clones were significantly decreased after the knockdown of CEP55 in KYSE520 and Eca109 cells (Figure 5(d)). To further confirm the result, we stain Ki-67 in ESCC tissues and a significant correlation was observed between Ki-67 expression and CEP55 (*r* = 0.28, *p*=0.049, Figure 5(e)).

### 3.7. CEP55 Contributes to ESCC Migration through the Src/FAK Pathway

**Figure 6:**
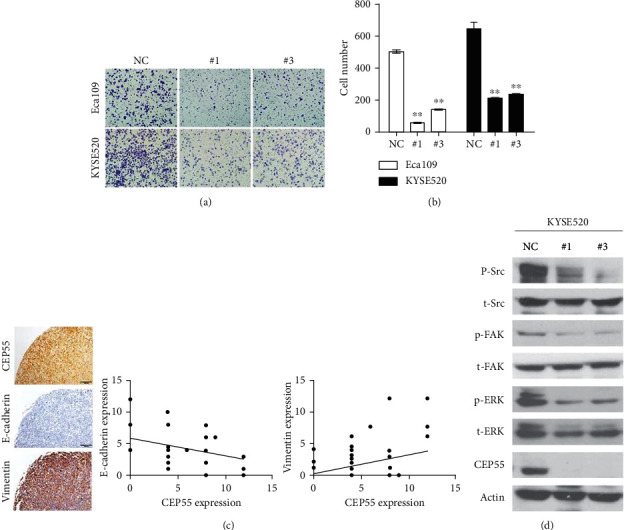
Knockdown of CEP55 suppresses cell migration in ESCC cell lines. (a and b) Eca109 and KYSE520 cells were transfected with NC or CEP55-RNAi and then suffered to transwell migration assay. And the number of migrated cells was counted. Data represent mean ± SD. Data presented as mean ± SD (^∗∗^*p* < 0.01). (c) A significant correlation was observed between E-cadherin expression and CEP55 expression (*r* = −0.299, *p*=0.035). Similar results were observed between vimentin and CEP55 expression (*r* = 0.289, *p*=0.042). (d) Downregulation of CEP-5 reduced phosphorylation of Src, FAK, and ERK were measured by western blotting assay.

According to our clinical data, CEPP55 is closely related to metastasis. Hence, to test whether CEP55 promotes ESCC cell metastasis in vitro, we examined the migration ability of KYSE520 and Eca109 cells by Transwell assay. We found that the number of migrated cells after siRNA transfection was dramatically reduced than after transfection with the mock control (NC) after 24 h of incubation (Figures 6(a) and 6(b)). To further confirm the result, we stain E-cadherin and vimentin in ESCC tissues. The results show that a significant correlation was observed between E-cadherin expression and CEP55 expression (*r* = −0.299, *p*=0.035). A similar result was observed between vimentin and CEP55 expression (*r* = 0.289, *p*=0.042), as shown in Figure 6(c).

Src/FAK signaling is known to be involved in cell migration. The Src and FAK complexes are nonreceptor tyrosine kinases, which are known to function in focal adhesion at sites where the extracellular matrix adhesion proteins interact with the cytoskeleton [22,23]. To investigate the mechanism by which CEP55 regulates cell migration, we examined the phosphorylation of Src and FAK. As shown in Figure 6(d), CEP55 silencing decreased the phosphorylation of FAK and Src. Moreover, ERK1/2, the downstream signaling effector of Src/FAK, was also inhibited by phosphorylation. This result indicated that CEP55 may reduce migration through inhibition of the Src/FAK pathway in ESCC cells.

## 4. Discussion

In this study, we showed that CEP55 is highly expressed in ESCC specimens and cell lines. Moreover, patients whose tumors have a high expression level of CEP55 have a poor outcome in terms of overall survival and disease-free survival. Additionally, a strong correlation was observed between CEP55 and TMN stage and between CEP55 and lymph node metastases in ESCC. Over the past decade, some studies have identified the role of CEP55 in many cancers such as breast cancer [18], colon cancer [19], and oral cavity squamous cell carcinoma [24] and found that this protein is involved in cell cycle regulation. Our study demonstrated that CEP55 promotes tumorigenesis of ESCC in vitro and revealed its role in ESCC cells.

We found that both the mRNA and protein levels of CEP55 were upregulated in ESCC samples as well as in cell lines. Moreover, a CEP55 expression profile in the Oncomine database further confirmed the credibility of our results. It is noteworthy that patients with tumors that expressed high levels of CEP55 had a lower OS and DFS than patients with tumors that expressed low levels of CEP55. A multivariate analysis also revealed that the level of CEP55 is a remarkable independent predictor of OS and DFS. Hence, CEP55 may be a prognostic indicator of ESCC.

According to previous reports, CEP55 promotes proliferation via a decrease in the number of cells in G2-M phase in different cancers [15]. Our results showed that the knockdown of CEP55 also inhibited colony formation of ESCC cell lines, which was similar to results from previous reports [15]. Our clinical data also showed that patients whose tumors had high CEP55 expression experienced more lymph node metastasis. This result indicated a possibility of a prometastasis function of CEP55 in ESCC. We tested the role of CEP55 in the migration ability of ESCC cells and found that the knockdown of CEP55 by siRNA transfection decreased the number of migrating cells compared with the control. These results revealed that CEP55 may be a marker of metastasis that directly promotes migration of ESCC cells. It is well-known that the Src/FAK pathway has an important function in cell migration through the reorganization of epithelial adhesion and the cytoskeleton [25]. FAK is a tyrosine kinase that is phosphorylated by ECM stimuli, and it further activates mitogen-activated proteins including ERK [26]. ERK mediates focal adhesion disassembly and translocates cellular signals from the cytoplasm to the nucleus, where it phosphorylates transcription factors that regulate cell migration [27–29]. Our data demonstrated that the knockdown of CEP55 decreased cell migration through the inhibition of the activities of Src, FAK, and ERK. Several studies have demonstrated that increased expression of these kinases has been associated with poor clinical outcome [30, 31]. As mentioned above, the clinical outcome of patients with high expression of CEP55 is associated with a poor prognosis and lymph node metastases. We also found increased phosphorylation of these kinases in vitro, which is in agreement with previous reports [30, 31]. However, larger-scale clinical data and functional in vivo experiments are still needed for further verification. A study conducted by Jia et al. investigated the prognostic value of CEP55 in pN0 ESCC and explored its biological function in ESCC cells [32]. They showed the dysregulation of PI3K/Akt pathway in CEP55 knock-down cells. Consistently, our results also showed that the knockdown of CEP55 significantly decreased the capability of proliferation, migration, invasion, and EMT processes of ESCC cells, but through different signaling pathways, including the inhibition of Src, FAK, and ERK. Therefore, our results deepen the understanding of CEP55's role in ESCC oncogenesis.

In conclusion, CEP55 is a promising biomarker that may not only be useful for early diagnosis but may also serve as a target gene for clinical therapeutic strategies.

## Figures and Tables

**Table 1:** CEP55 expression and clinicopathologic variables in ESCC patients.

Variable	CEP55 expression			
	Cases (*n* = 241)	Low (*n* = 99, percent)	High (*n* = 142, percent)	*p* value^a^
Age^b^ (years)				0.155
≤58	128	58 (45.3)	70 (54.7)	
>58	113	41 (36.3)	72 (63.7)	

Sex				0.739
Male	63	27 (142.9)	36 (57.1)	
Female	178	72 (40.4)	106 (59.60	

Tumor location				0.639
Upper ESCC	12	4 (33.3)	8 (66.7)	
Middle ESCC	165	71 (43.0)	94 (57.0)	
Lower ESCC	64	24 (37.5)	40 (62.5)	

Histological grade				0.273
Grade 1	58	29 (50.0)	29 (50.0)	
Grade 2	155	60 (38.7)	95 (61.3)	
Grade 2	64	10 (35.7)	18 (64.3)	

pT status				0.869
pT 1	6	3 (50.0)	3 (50.0)	
pT 2	54	20 (37.00)	34 (63.0)	
pT 3	178	75 (42.1)	103 (57.9)	
pT 4	3	1 (33.3)	2 (66.7)	

N categories				0.024
Negative	130	62 (47.7)	68 (52.3)	
Positive	111	37 (33.3)	74 (66.7)	

TNM stage				0.046
Stage I	9	5 (55.6)	4 (44.4)	
Stage II	138	64 (46.4)	74 (53.6)	
Stage III	93	29 (31.2)	64 (68.8)	

^a^Probability value of <0.05 indicates statistical significance. Probability values are calculated by Pearson's c2 test. ^b^Age is divided according to the median age of 58 years. ^c^The grading and histopathology stage of ESCC specimens are based on the World Health Organization (WHO) classification published in 2009.

**Table 2:** Analysis of risk factors for overall survival in ESCC patients.

Variable	Univariate analysis		Multivariate analysis	
	HR (95% CI)	*p* value	HR (95% CI)	*p* value
Age b (years)		0.779		
≤58	1.000			
>58	0.951 (0.671–1.349)			

Sex		0.162		
Male	1.000			
Female	1.340 (0.889–2.020)			

Tumor location		0.325		
Upper ESCC	1.000			
Middle ESCC	1.628 (0.777–1.536)			
Lower ESCC	0.968 (0.644–1.454)			

Histological grade		0.130		
Grade 1	1.000			
Grade 2	1.007 (0.660–1.536)			
Grade 2	1.656 (0.931–2.948)			

pT status		<0.001		<0.001
pT 1	1.000		1.000	
pT 2	1.596 (0.377–6.760)		1.726 (0.407–7.324)	
pT 3	2.272 (0.559–9.231)		2.308 (0.567–9.403)	
pT 4	64.653 (9.871–423.451)		91.578 (13.736–610.554)	

N categories		<0.001		<0.001
Negative	1.000		1.000	
Positive	3.457 (2.381–5.018)		3.280 (2.245–4.791)	

TNM stage		<0.001		
Stage I	1.000			
Stage II	1.047 (0.326–3.357)			
Stage III	4.158 (1.304–13.261)			

CEP55 expression		<0.001		0.009
Low	1.000		1.000	
High	1.960 (1.340–2.865)		1.669 (1.136–2.453)	

**Table 3:** Analysis for risk factors for disease-free survival in ESCC patients.

Variable	Univariate analysis		Multivariate analysis	
	HR (95% CI)	*p* value	HR (95% CI)	*p* value
Age b (years)		0.824		
≤58	1.000			
>58	0.961 (0.678–1.363)			

Sex		0.218		
Male	1.000			
Female	1.294 (0.859–1.951)			

Tumor location		0.348		
Upper ESCC	1.000			
Middle ESCC	1.663 (0.793–3.487)			
Lower ESCC	1.015 (0.676–1.525)			

Histological grade		0.076		
Grade 1	1.000			
Grade 2	1.020 (0.669–1.555)			
Grade 2	1.775 (0.998–3.156)			

pT status		<0.001		<0.001
pT 1	1.000		1.000	
pT 2	1.439 (0.340–6.091)		1.444 (0.340–6.120)	
pT 3	1.983 (0.488–8.051)		1.866 (0.459–7.596)	
pT 4	30.493 (4.752–195.655)		37.674 (5.823–243.733)	

N categories		<0.001		<0.001
Negative	1.000		1.000	
Positive	3.319 (2.287–4.815)		3.115 (2.135–4.545)	

TNM stage		<0.001		
Stage I	1.000			
Stage II	1.066 (0.332–3.419)			
Stage III	3.952 (1.241–12.586)			

CEP55 expression		0.001		0.015
Low	1.000		1.000	
High	1.894 (1.296–2.768)		1.612 (1.097–2.369)	

## Data Availability

The data used to support this study are deposited in Research Data Deposit (RDDB2021001603).

## Supplementary Materials

Supplementary Materials Supplementary Figure 1: immunohistochemical staining of CEP55 in human ESCC samples. (A and B) negative expression of CEP55 in normal esophageal squamous epithelium tissue sever as negative control. (A) 200x, (B) 400x. (C and D) negative expression of CEP55 in ESCC. (C) 200x, (D) 400x. (E and F) weak staining of CEP55 in cytoplasm. (E) 200x, (F) 400x. (G and H) moderate expression of CEP55 in cytoplasm. (G) 200x, (H) 400x. (I and J) strong staining of CEP55 in cytoplasm. (I) 200x, (J) 400x. Supplementary Figure 2: selection of the cutoff value for the CEP55 Immunoreactivity score. Receiver operating curve for CEP55 expression cutoff value was plotted by survival status.
