# Supplementary figures and images for: CEP55 Positively Affects Tumorigenesis of Esophageal Squamous Cell Carcinoma and Is Correlated with Poor Prognosis
Source: J Oncol. 2021 May 18;2021:8890715. doi: 10.1155/2021/8890715 (PMC8159646; doi:10.1155/2021/8890715)

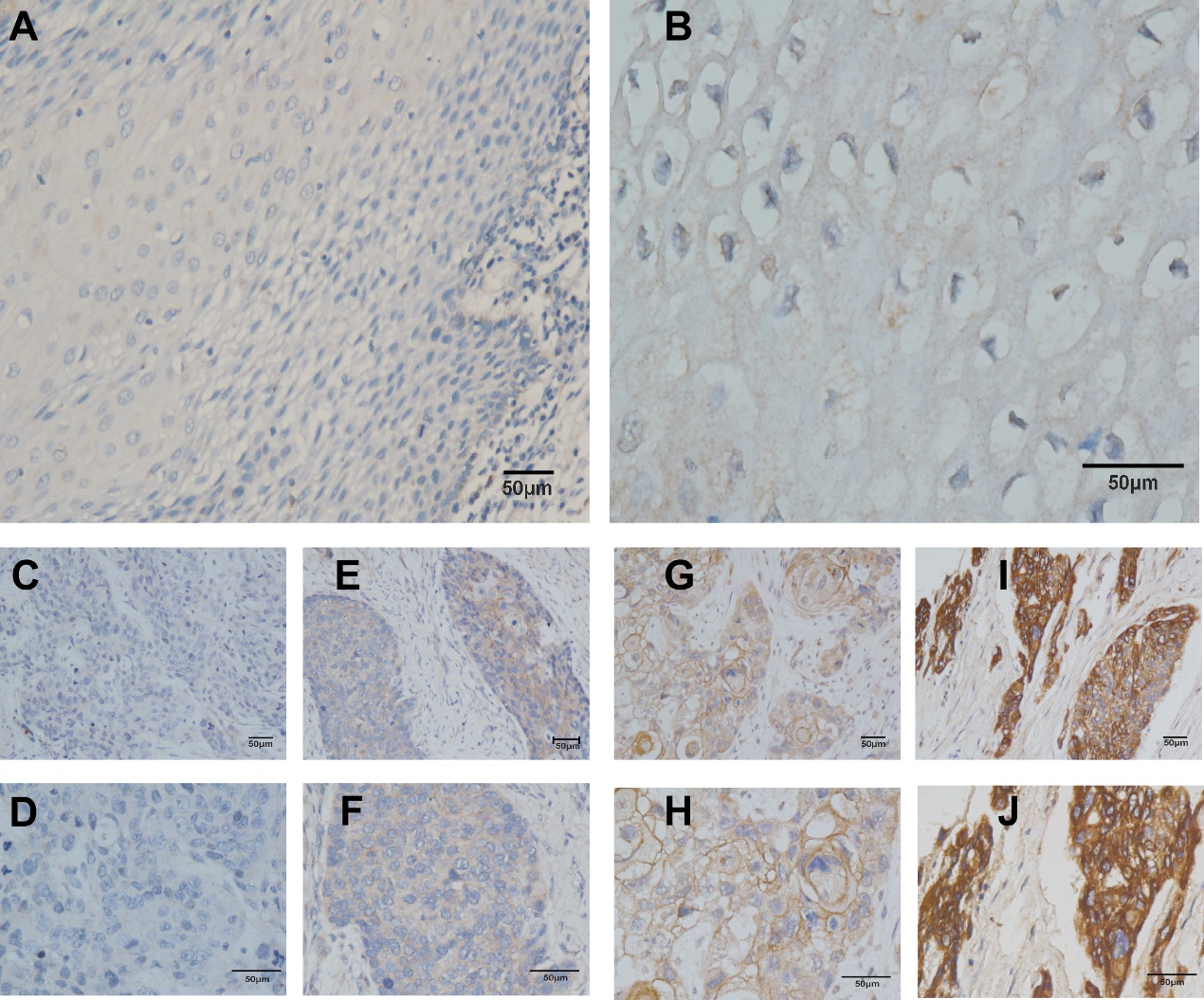


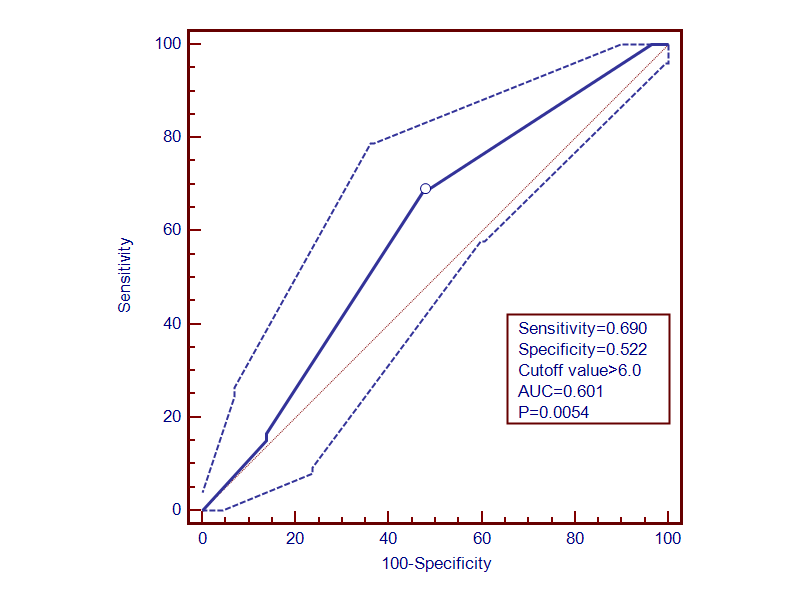

Supplement: Supplementary Materials — Supplementary Figure 1: immunohistochemical staining of CEP55 in human ESCC samples. (A and B) negative expression of CEP55 in normal esophageal squamous epithelium tissue sever as negative control. (A) 200x, (B) 400x. (C and D) negative expression of CEP55 in ESCC. (C) 200x, (D) 400x. (E and F) weak staining of CEP55 in cytoplasm. (E) 200x, (F) 400x. (G and H) moderate expression of CEP55 in cytoplasm. (G) 200x, (H) 400x. (I and J) strong staining of CEP55 in cytoplasm. (I) 200x, (J) 400x. Supplementary Figure 2: selection of the cutoff value for the CEP55 Immunoreactivity score. Receiver operating curve for CEP55 expression cutoff value was plotted by survival status. [file 8890715.f1.docx]
